# Supplementary material for: Effect of TiO2 Film Thickness on the Stability of Au9 Clusters with a CrOx Layer
Source: Nanomaterials (Basel). 2022 Sep 16;12(18):3218. doi: 10.3390/nano12183218 (PMC9506353; doi:10.3390/nano12183218)
Supplement: Supplementary file 1 [file nanomaterials-12-03218-s001.zip › nanomaterials-1895874-supplementary.pdf]

Supporting Information for:

Effect of TiO_2 Film Thickness on the Stability of Au_9 Clusters with a CrO_x layer

Abdulrahman S. Alotabi [1,2,3], Yanting Yin[1,3], Ahmad Redaa[4,5], Siriluck Tesana[6,7],

Gregory F. Metha[8,]* and Gunther G. Andersson[1,3,]*

[1] Flinders Institute for Nanoscale Science and Technology, Flinders University, Adelaide, South Australia 5042, Australia
[2] Department of Physics, Faculty of Science and Arts in Baljurashi, Albaha University, Baljurashi 65655, Saudi Arabia
[3] Flinders Microscopy and Microanalysis, College of Science and Engineering, Flinders University, Adelaide, South Australia 5042, Australia
[4] Department of Earth Sciences, University of Adelaide, Adelaide, South Australia 5005, Australia
[5] Faculty of Earth Sciences, King Abdulaziz University, Jeddah, Saudi Arabia
[6] The MacDiarmid Institute for Advanced Materials and Nanotechnology,
School of Physical and Chemical Sciences, University of Canterbury, Christchurch
8041, New Zealand
[7] National Isotope Centre, GNS Science, Lower Hutt 5010, New Zealand
[8] Department of Chemistry, University of Adelaide, Adelaide, South Australia 5005, Australia

* **Corresponding authors:** Gunther G. Andersson, Gregory F. Metha

Email: gunther.andersson@flinders.edu.au, greg.metha@adelaide.edu.au.

Address: Physical Sciences Building (2111) GPO Box 2100, Adelaide 5001, South Australia

This supporting information document contains detailed descriptions about each of the applied characterization techniques, and also presents the following additional figures and tables:

Figure S1: *A photo of the TiO_2P (lift) and TiO_2G (right) films.*

Figure S2: *UV-Vis spectrum of $Au_9(PPh_3)_8(NO_3)_3$ in Methanol.*

Figure S3*: Cross-section SEM-EDAX elemental maps of Ti, O and Si of TiO_2P and TiO_2G. Note that the scale bars are different.*

Figure S4: *3D Profile of **(A)** TiO_2P before heating, **(B)** TiO_2P after heating, **(C)** before heating, TiO_2G and **(D)** TiO_2G after heating (area 16 × 16 µm).*

Figure S5*: Surface morphology with the average of Ra and Rq values of **(A)** TiO_2P before heating, **(B)** TiO_2P after heating, **(C)** before heating, TiO_2G and **(D)** TiO_2G after heating. (area 595 × 595 µm). It is important to know that the scale bars are different.*

Figure S6: *XP spectra of Au 4f of **(A)** TiO_2P-Au_9: after Au_9 deposition (blue) and after heating (grey) **(B)** TiO_2P-Au_9-CrO_x: after Au_9 deposition (blue), after CrO_x layer photodeposited (orange) and after heating (grey).*

Figure S7: *XP spectra of Au 4f of **(A)** TiO_2G-Au_9: after Au_9 deposition (blue) and after heating (grey) **(B)** TiO_2G-Au_9-CrO_x: after Au_9 deposition (blue), after CrO_x layer photodeposited (orange) and after heating (grey)*

Figure S8: *XP spectra of P 2p of **(A)** TiO_2P-Au_9: after Au_9 deposition (blue) and after heating (grey) **(B)** TiO_2P-Au_9-CrO_x: after Au_9 deposition (blue), after CrO_x layer photodeposited (orange), and after heating (grey)*

Figure S9: *XP spectra of Cr 2p of the TiO_2P-Au_9-CrO_x sample of **(A)** 0.006mM sample, **(B)** 0.06mM sample and **(C)** 0.6mM sample: after CrO_x layer photodeposited (orange) and after heating (grey).*

Figure S10: *XP spectra of P 2p of **(A)** TiO_2G-Au_9: after Au_9 deposition (blue) and after heating (grey) **(B)** TiO_2G-Au_9-CrO_x: after Au_9 deposition (blue), after CrO_x layer photodeposited (orange), and after heating (grey)*

Figure S11: *XP spectra of Cr 2p of the TiO_2G-Au_9-CrO_x sample of **(A)** 0.006mM sample, **(B)** 0.06mM sample and **(C)** 0.6mM sample: after CrO_x layer photodeposited (orange) and after heating (grey).*

Table S1*: XPS Au $4f_{7/2}$ peak positions and FWHM of TiO_2P-Au_9 and TiO_2P-Au_9-CrO_x.*

Table S2*: XPS Au $4f_{7/2}$ peak positions and FWHM of TiO_2G-Au_9 and TiO_2G-Au_9-CrO_x.*

Table S3*: XPS Cr $2p_{3/2}$ peak positions and FWHM of TiO_2P.*

Table S4*: XPS Cr $2p_{3/2}$ peak positions and FWHM of TiO_2G.*

Table S5*: XPS relative amount of Cr $2p_{3/2}$ to Ti $2p_{3/2}$ of TiO_2P-Au_9-CrO_x and TiO_2G-Au_9-CrO_x.*

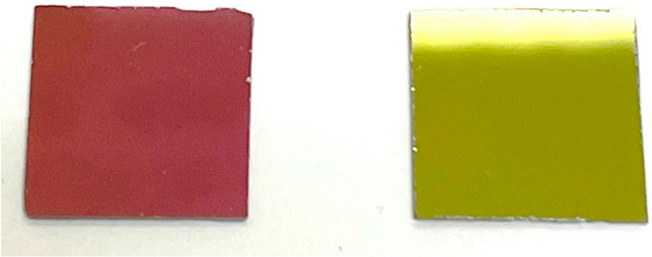

Figure S1: *A photo of the TiO_2P (lift) and TiO_2G (right) films.*

Figure S2 shows the UV-Vis spectrum of synthesized $Au_9(PPh_3)_8(NO_3)_3$ clusters with four peaks around 315, 350, 375 and 440 nm. The UV-Vis spectrum is in agreement with those obtained from literatures of $Au_9(PPh_3)_8(NO_3)_3$, confirming the synthesis of Au_9 clusters[1,2].

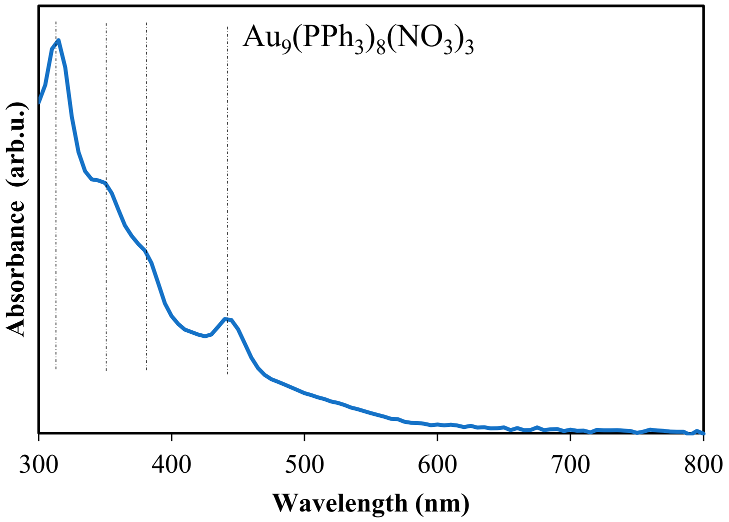

Figure S2: *UV-Vis spectrum of $Au_9(PPh_3)_8(NO_3)_3$ in Methanol.*

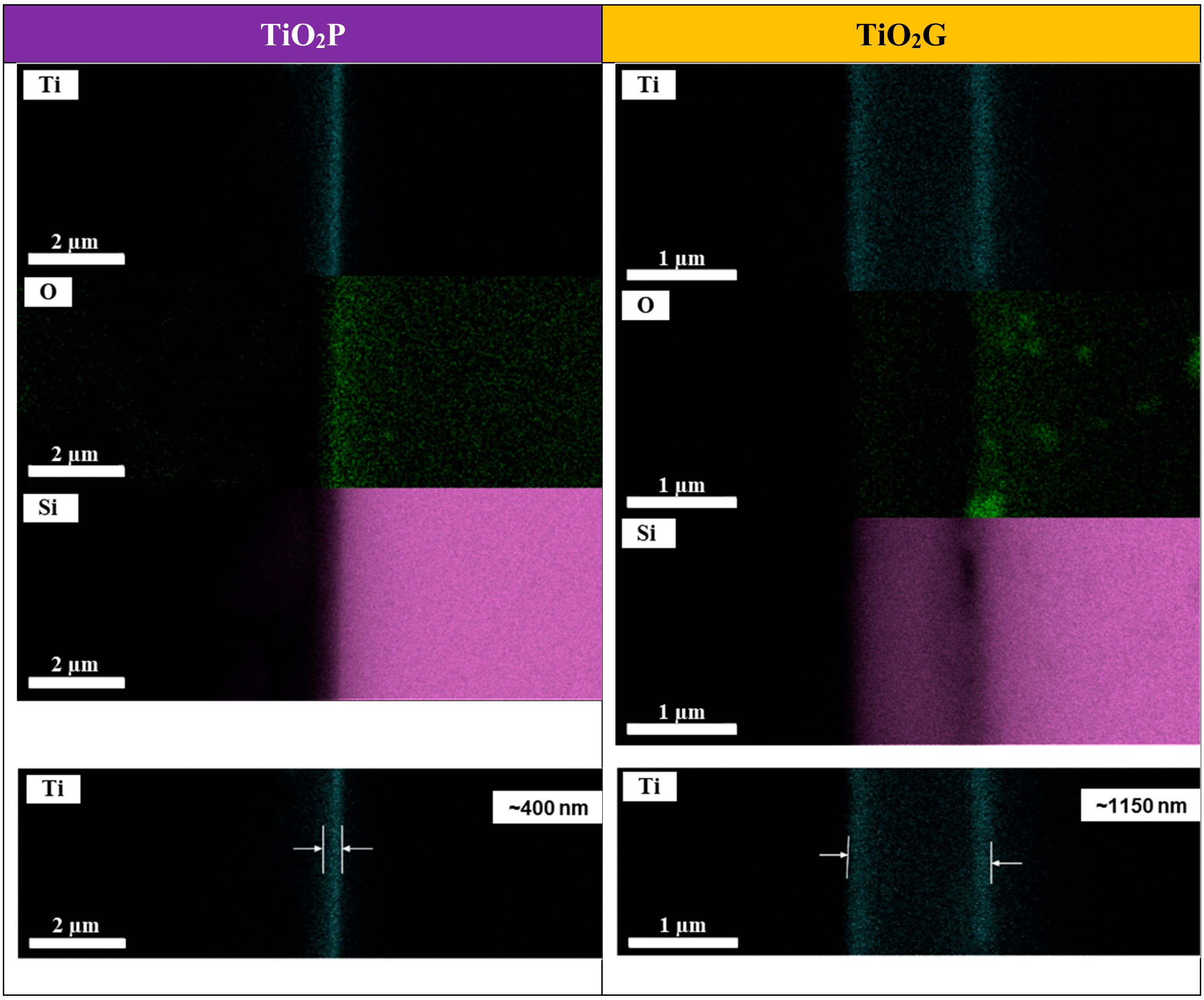

***Figure S3**: Cross-section SEM-EDAX elemental maps of Ti, O and Si of TiO_2P and TiO_2G. Note that the scale bars are different.*

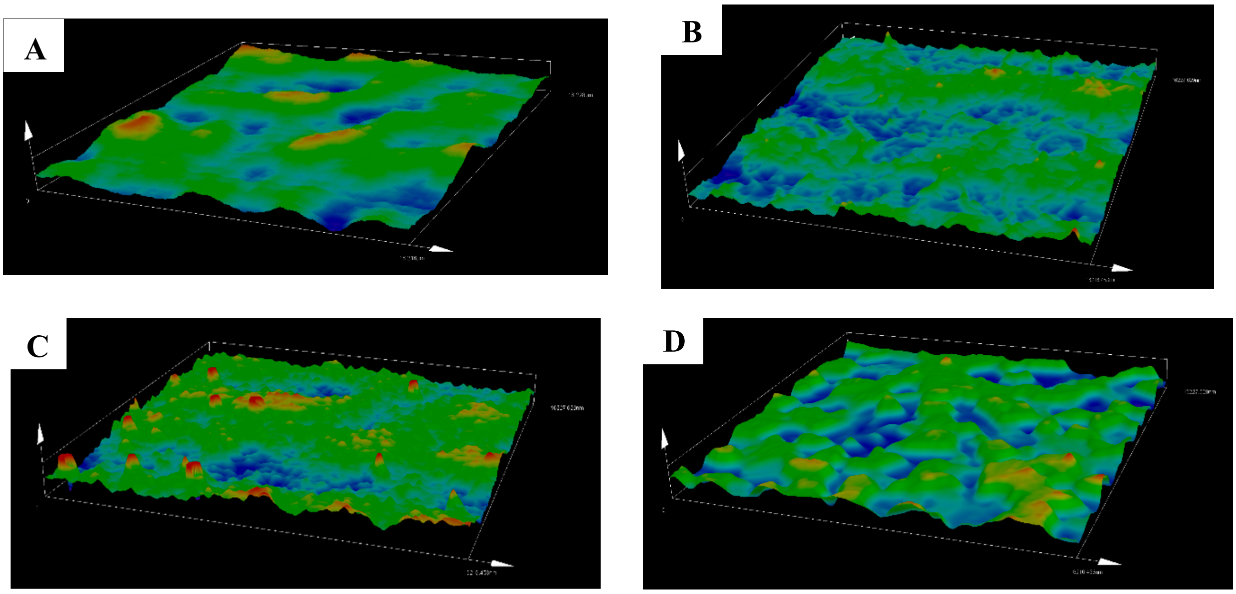

Figure S4: *3D Profile of (**A**) TiO_2P before heating, (**B**) TiO_2P after heating, (**C**) before heating, TiO_2G and (**D**) TiO_2G after heating (area 16 × 16 µm).*

Figure S5 shows the average Ra and Rq values with a large area scan 595 × 595 µm of TiO_2P and TiO_2G before and after heating. For TiO2P before heating, the Ra and Rq values are 1.4 nm and 1.8 nm, while after heating are 1.6 nm and 2.1 nm. For TiO2G before heating, the Ra and Rq values are 2.8 nm and 4.6 nm, while after heating are 15.0 nm and 17.4 nm. The change in the Ra and Rq values after heating are 0.2 nm and 0.3 nm. for the thin TiO_2 layer (TiO_2P), however, the thick TiO_2 layer (TiO_2G) are 12.2 nm and 12.8 nm. The Ra and Rq values of TiO_2G after heating are 12 times higher compared to before heating. The change in the surface morphology of TiO_2 films after heating is due to the transformation of TiO_2 films from amorphous to anatase[3-5]. Çörekçí et al, showed that the change in the surface morphology of TiO_2 film is higher for the thicker film than a thin film after heating. This due to the high mobility (recrystallisation) of the thicker film during the thermal heating[4]. Here, the significant change in the morphology of TiO_2G after heating is due to the high mobility of TiO_2G during heating, which is affected by the transformation to anatase phase.

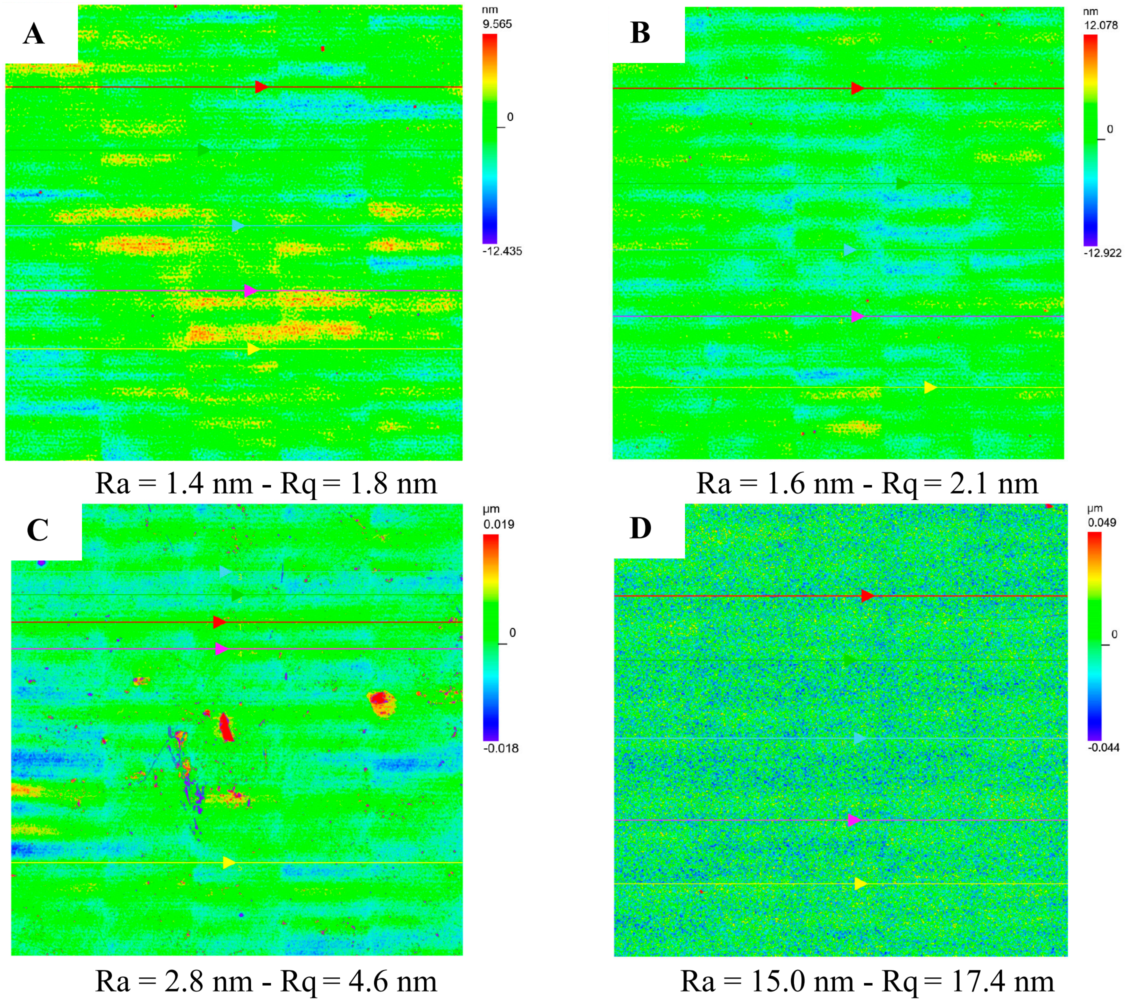

***Figure S5**: Surface morphology with the average of Ra and Rq values of (**A**) TiO_2P before heating, (**B**) TiO_2P after heating, (**C**) before heating, TiO_2G and (**D**) TiO_2G after heating. (area 595 × 595 µm). It is important to know that the scale bars are different.*

***Table S1**: XPS Au $4f_{7/2}$ peak positions and FWHM of TiO_2P-Au_9 and TiO_2P-Au_9-CrO_x.*

	Before heating		After CrO_x photodeposition		After heating	
Au_9 concentration	Peak position (eV)	FWHM	Peak position (eV)	FWHM	Peak position (eV)	FWHM
Without CrO_x layer						
0.006 mM	85.1 ± 0.2	1.7 ± 0.2	-	-	84.8 ± 0.2	1.5 ± 0.2
0.06 mM	85.4 ± 0.2	1.7 ± 0.2	-	-	84.8 ± 0.2	1.6 ± 0.2
0.6 mM	85.1 ± 0.2	1.8 ± 0.2	-	-	84.7 ± 0.2	1.6 ± 0.2
With CrO_x layer						
0.006 mM	85.2 ± 0.2	1.8 ± 0.2	85.3 ± 0.2	1.6 ± 0.2	85.0 ± 0.2	1.7 ± 0.2
0.06 mM	85.1 ± 0.2	1.8 ± 0.2	85.3 ± 0.2	1.6 ± 0.2	85.0 ± 0.2	1.7 ± 0.2
0.6 mM	85.1 ± 0.2	1.8 ± 0.2	85.3 ± 0.2	1.6 ± 0.2	85.0 ± 0.2	1.7 ± 0.2

***Table S2**: XPS Au $4f_{7/2}$ peak positions and FWHM of TiO_2G-Au_9 and TiO_2G-Au_9-CrO_x.*

	Before heating		After CrO_x photodeposition		After heating	
Au_9 concentration	Peak position (eV)	FWHM	Peak position (eV)	FWHM	Peak position (eV)	FWHM
**Without CrO_x layer**						
0.006 mM	85.4 ± 0.2	1.8 ± 0.2	-	-	84.9 ± 0.2	1.7 ± 0.2
0.06 mM	85.4 ± 0.2	1.8 ± 0.2	-	-	84.6 ± 0.2	1.6 ± 0.2
0.6 mM	85.3 ± 0.2	1.8 ± 0.2	-	-	84.6 ± 0.2	1.5 ± 0.2
**With CrO_x layer**						
0.006 mM	85.3 ± 0.2	1.6 ± 0.2	85.5 ± 0.2	1.6 ± 0.2	84.8 ± 0.2	1.8 ± 0.2
0.06 mM	85.2 ± 0.2	1.6 ± 0.2	85.5 ± 0.2	1.6 ± 0.2	84.4 ± 0.2	1.6 ± 0.2
0.6 mM	84.9 ± 0.2	1.8 ± 0.2	85.3 ± 0.2	1.6 ± 0.2	84.3 ± 0.2	1.3 ± 0.2

Figure S6 shows the Au 4f spectra of TiO_2P-Au_9 and TiO_2P-Au_9-CrO_x. The blue line is after the Au_9 cluster deposition, orange line is after the photodeposition of CrO_x layer and grey line is after heating at 200°C for 10min to remove the Au_9 clusters ligands. The black lines indicate the binding energy at 85 eV.

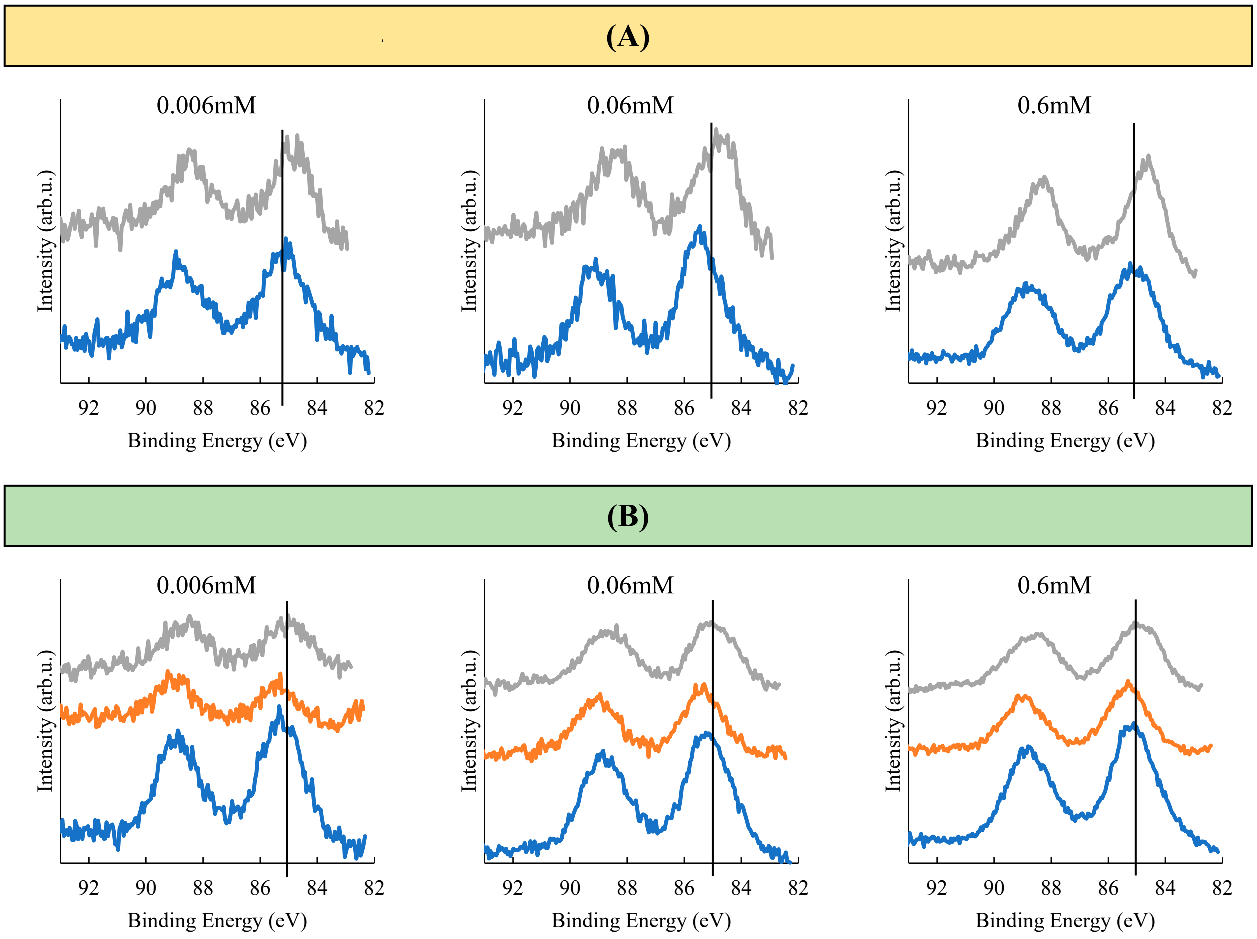

***Figure S6:** XP spectra of Au 4f of **(A)** TiO_2P-Au_9: after Au_9 deposition (blue) and after heating (grey) **(B)** TiO_2P-Au_9-CrO_x: after Au_9 deposition (blue), after CrO_x layer photodeposited (orange) and after heating (grey).*

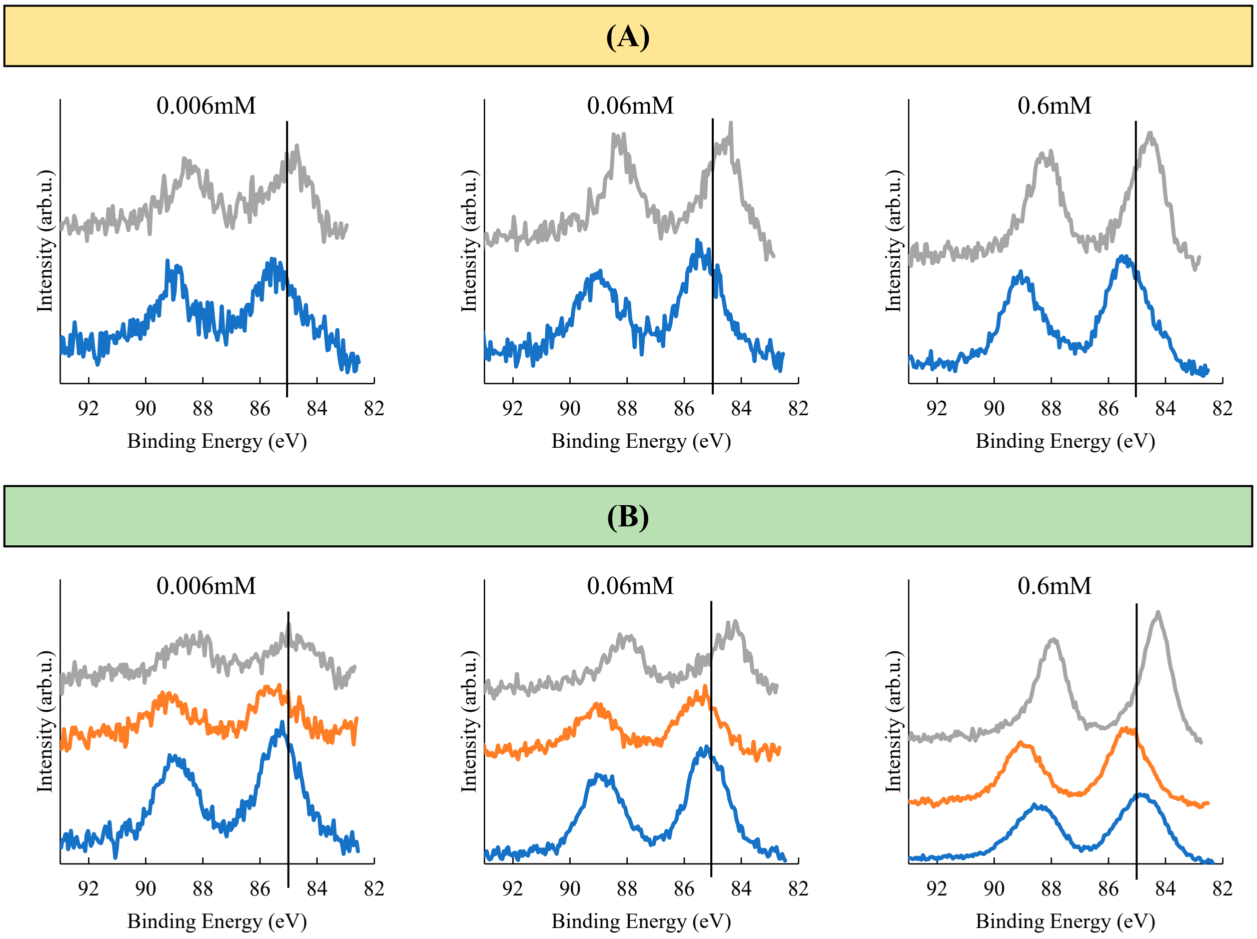

***Figure S7:** XP spectra of Au 4f of **(A)** TiO_2G-Au_9: after Au_9 deposition (blue) and after heating (grey) **(B)** TiO_2G-Au_9-CrO_x: after Au_9 deposition (blue), after CrO_x layer photodeposited (orange) and after heating (grey)*

Figure S8 shows the P 2p spectra of TiO_2P-Au_9 and TiO_2P-Au_9-CrO_x. The blue line is after the Au_9 cluster deposition, orange line is after the photodeposition of CrO_x layer and grey line is after heating. The black lines indicate the position of triphenylphosphine ligands at 131.8 eV[6,7]. The P 2p spectrum can be fitted with doublet ($2p_{3/2}$ and $2p_{1/2}$), the splitting was fixed at 0.84 eV. Before heating, for 0.6 mM, the P $2p_{3/2}$ peak is appeared at 131.8 ± 0.2 eV, which relates to the peak position of PPh_3 ligands bounds to Au_9 clusters[6,7]. After heating, no peak is detected, indicating that phosphorous ligands were removed by heating for 0.6 mM. For the lower concentrations of 0.06 and 0.006 mM

with the addition of the CrO_x overlayer, the P 2p signal is below the detection limit. However, the same phenomenon of ligand removal via heating is expected for these lower concentrations [8,9]. The size of Au_9 clusters were more stable using the laboratory based XPS instrument comparing to synchrotron XPS. This assumed to be due to ageing of the Au_9 clusters with the Synchrotron XPS results, where the samples were measured a week after the deposition of Au_9 and the photodeposition of CrO_x[10]. While using the laboratory based XPS instrument, the measurements were performed directly after the deposition of Au_9 and followed by photodeposition of CrO_x layer. The conclusion is similar to that reported previously from synchrotron data that the agglomeration of Au_9 clusters was inhibited with CrO_x layer.

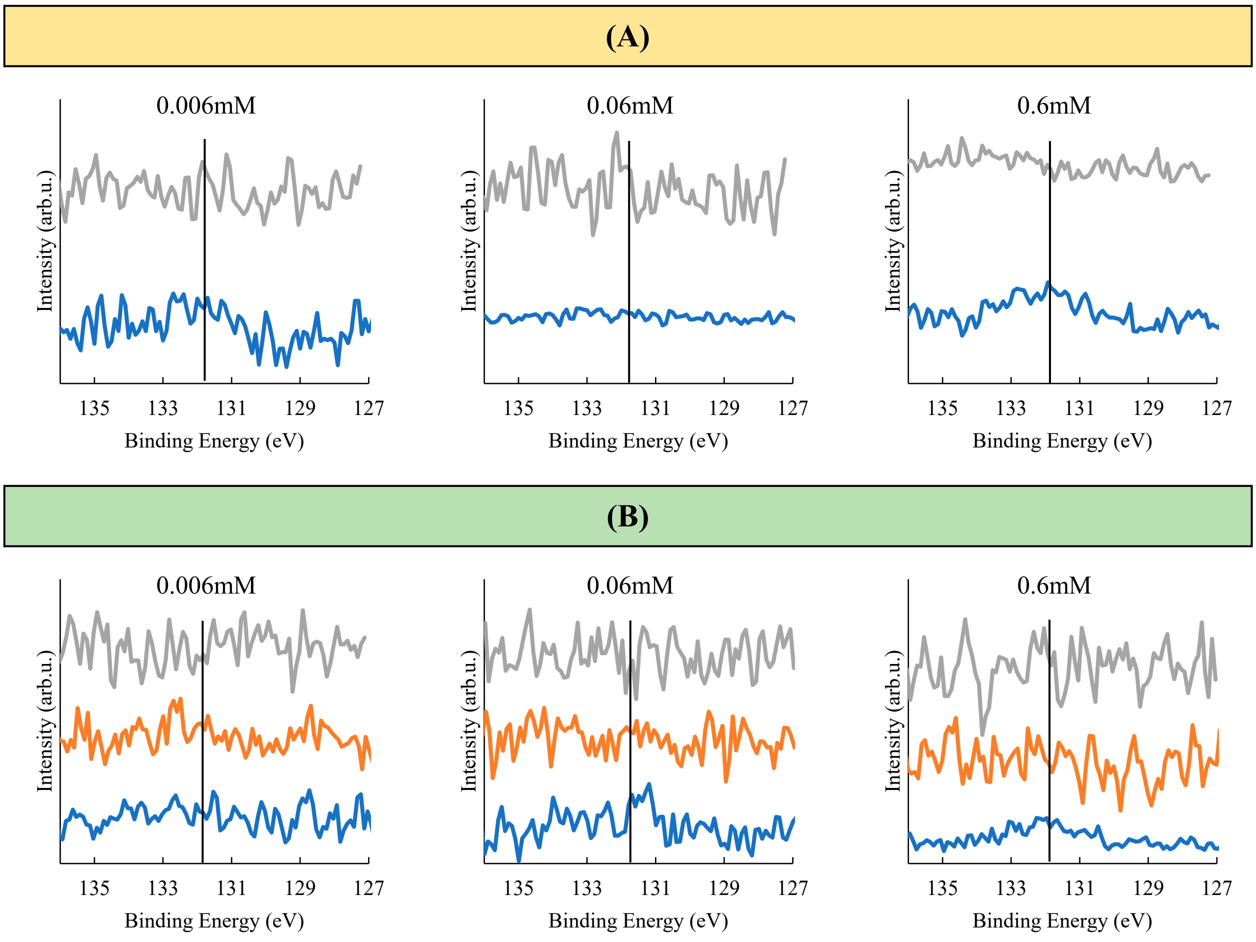

Figure S8: *XP spectra of P 2p of* ***(A)*** *TiO_2P-Au_9: after Au_9 deposition (blue) and after heating (grey)* ***(B)*** *TiO_2P-Au_9-CrO_x: after Au_9 deposition (blue), after CrO_x layer photodeposited (orange), and after heating (grey)*

Figure S9 shows the Cr 2p spectra of TiO_2P-Au_9 and TiO_2P-Au_9-CrO_x. Before heating, the Cr $2p_{3/2}$ peak is appeared at 577.7 ± 0.2 eV, which relates to the peak position of $Cr(OH)_3$[11]. After heating, the Cr $2p_{3/2}$ peak position shifts to a low binding energy at 577.1 ± 0.2 eV, which corresponds to Cr_2O_3[12,13]. This confirms that the CrO_x layer is reduced by heating from $Cr(OH)_3$ to Cr_2O_3. This in agreement to previous studies report that heating reduces $Cr(OH)_3$ layer to Cr_2O_3 [14].

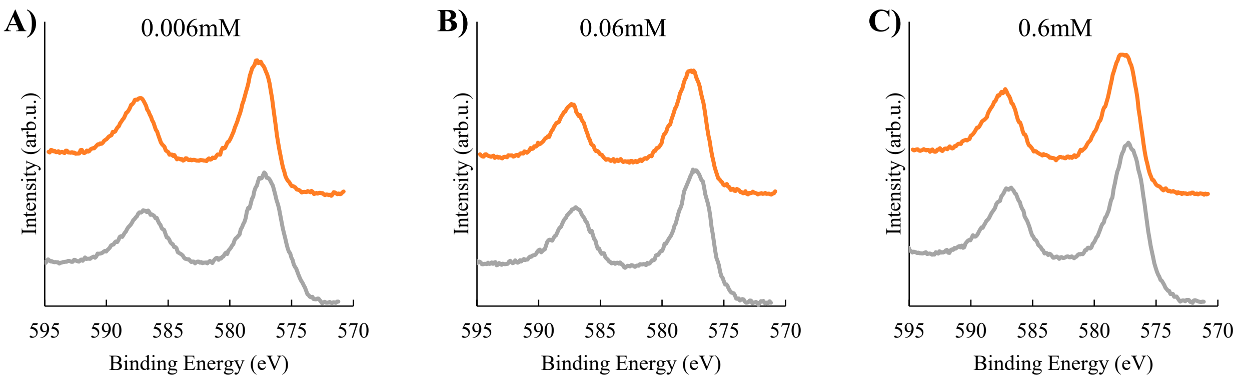

Figure S9: *XP spectra of Cr 2p of the TiO_2P-Au_9-CrO_x sample of **(A)** 0.006mM sample, **(B)** 0.06mM sample and **(C)** 0.6mM sample: after CrO_x layer photodeposited (orange) and after heating (grey).*

Table S3*: XPS Cr $2p_{3/2}$ peak positions and FWHM of TiO_2P-Au_9-CrO_x.*

	TiO_2P			
	Before heating		After heating	
	Peak position (eV)	FWHM	Peak position (eV)	FWHM
0.006 mM	577.7 ± 0.2	2.7 ± 0.2	577.1 ± 0.2	3.4 ± 0.2
0.06 mM	577.7 ± 0.2	2.9 ± 0.2	577.1 ± 0.2	3.0 ± 0.2
0.6 mM	577.7 ± 0.2	2.9 ± 0.2	577.2 ± 0.2	3.0 ± 0.2

Figure S10 shows the P 2p spectra of TiO_2G-Au_9 without and with CrO_x layer before and after heating. For 0.6 mM, the P $2p_{3/2}$ peak before heating are found at 131.8 ± 0.2 eV for both without and with CrO_x layer, referring to the peak of PPh_3 ligands[6,7]. After heating, the P $2p_{3/2}$ peak is found at 133.7 ± 0.2 eV, which attribute to removed ligands from the Au_9 clusters and oxidized by attaching to TiO_2 substrate as previously reported[6,9]. For 0.06 and 0.006 mM, the signal of the P 2p is below the detection limit but it is expected that same behavior occurs for these lower concentrations.

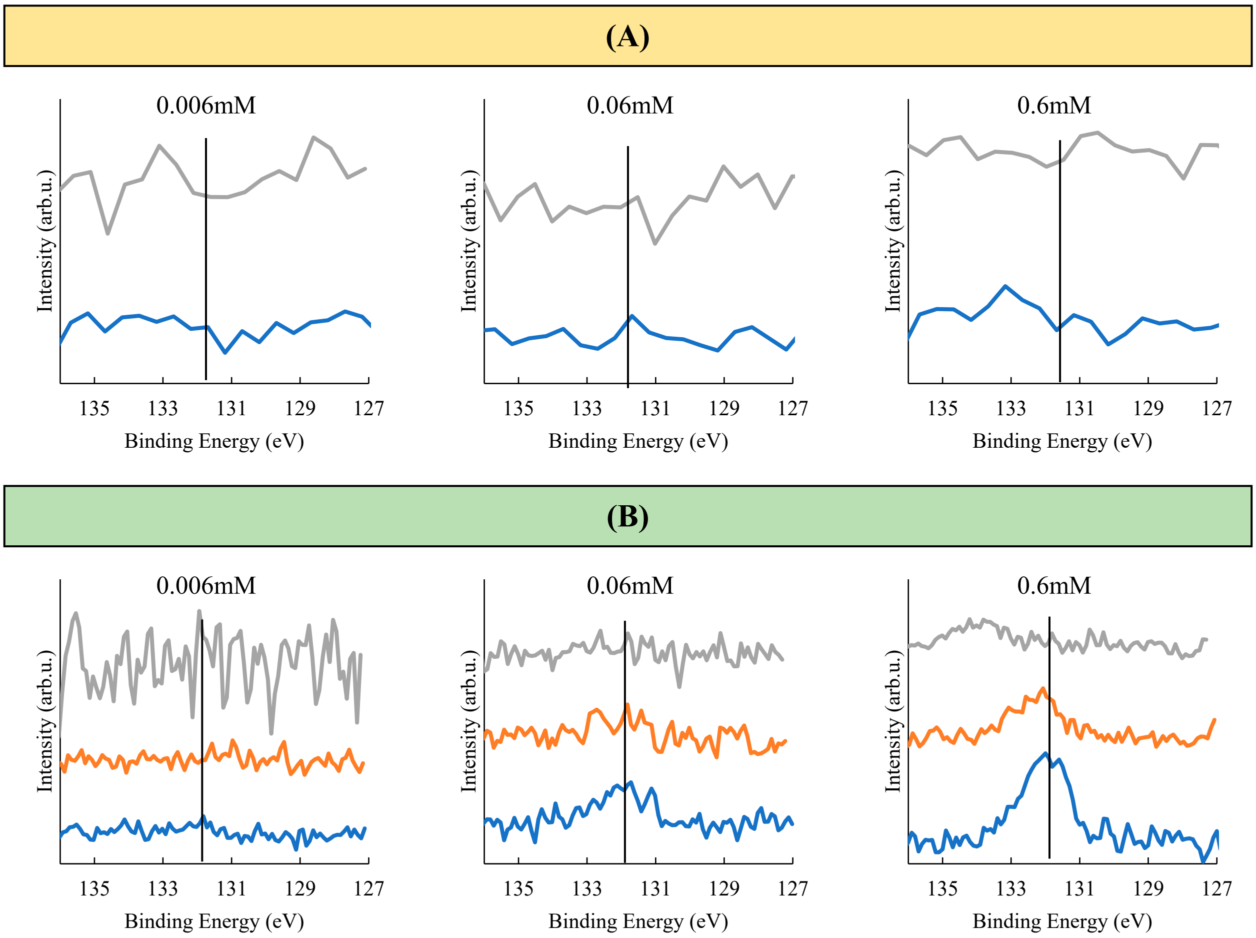

Figure S10: *XP spectra of P 2p of* ***(A)*** *TiO_2G-Au_9: after Au_9 deposition (blue) and after heating (grey)* ***(B)*** *TiO_2G-Au_9-CrO_x: after Au_9 deposition (blue), after CrO_x layer photodeposited (orange), and after heating (grey)*

Figure S11 shows the Cr 2p spectra of TiO_2G-Au_9 and TiO_2G-Au_9-CrO_x. The Cr $2p_{3/2}$ peak is appeared at 577.8 ± 0.2 eV before heating for all samples. This binding energy is similar to that of $Cr(OH)_3$[11]. The peak position of Cr $2p_{3/2}$ shifts to a low binding energy at 577.3 ± 0.2 eV after

heating, which relates to the binding energy of Cr_2O_3 [13]. This in consistent with TiO_2P substrate that CrO_x layer is reduced from $Cr(OH)_3$ to Cr_2O_3 by heating.

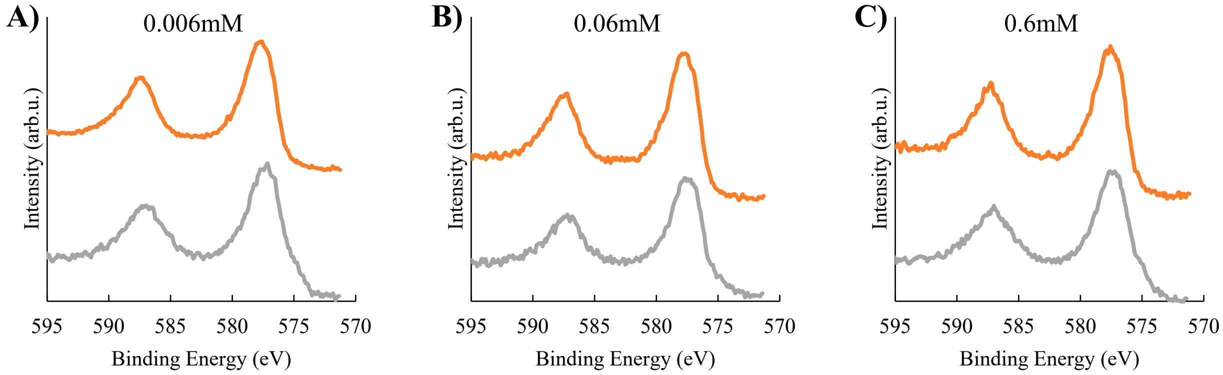

Figure S11: *XP spectra of Cr 2p of the TiO_2G-Au_9-CrO_x sample of* ***(A)*** *0.006mM sample,* ***(B)*** *0.06mM sample and* ***(C)*** *0.6mM sample: after CrO_x layer photodeposited (orange) and after heating (grey).*

Table S4*: XPS Cr $2p_{3/2}$ peak positions and FWHM of TiO_2G-Au_9-CrO_x.*

	**TiO_2G**			
	Before heating		After heating	
	Peak position (eV)	FWHM	Peak position (eV)	FWHM
0.006 mM	577.8 ± 0.2	2.8 ± 0.2	577.3 ± 0.2	3.3 ± 0.2
0.06 mM	577.8 ± 0.2	2.8 ± 0.2	577.5 ± 0.2	2.8 ± 0.2
0.6 mM	577.6 ± 0.2	2.7 ± 0.2	577.3 ± 0.2	3.0 ± 0.2

Table S5*: XPS relative amount of Cr $2p_{3/2}$ to Ti $2p_{3/2}$ of TiO_2P-Au_9-CrO_x and TiO_2G-Au_9-CrO_x.*

	**Cr/Ti**					
	**TiO_2P-Au_9-CrO_x**			**TiO_2G-Au_9-CrO_x**		
	Before heating	After heating	Difference %	Before heating	After heating	Difference %
0.006 mM	2.74	1.47	47	1.48	0.62	58
0.06 mM	1.54	1.14	26	1.30	0.40	69
0.6 mM	3.12	1.99	36	1.82	0.72	61

REFERENCES

1. Adnan, R.H.; Madridejos, J.M.L.; Alotabi, A.S.; Metha, G.F.; Andersson, G.G. A Review of State of the Art in Phosphine Ligated Gold Clusters and Application in Catalysis. *Advanced Science* **2022**, *9*, 2105692, doi:https://doi.org/10.1002/advs.202105692.
2. Madridejos, J.M.L.; Harada, T.; Falcinella, A.J.; Small, T.D.; Golovko, V.B.; Andersson, G.G.; Metha, G.F.; Kee, T.W. Optical Properties of the Atomically Precise C4 Core [Au9(PPh3)8]3+ Cluster Probed by Transient Absorption Spectroscopy and Time-Dependent Density Functional Theory. *The Journal of Physical Chemistry C* **2021**, *125*, 2033-2044, doi:10.1021/acs.jpcc.0c08838.
3. Mathews, N.R.; Morales, E.R.; Cortés-Jacome, M.A.; Toledo Antonio, J.A. TiO2 Thin Films – Influence of Annealing Temperature on Structural, Optical and Photocatalytic Properties. *Solar Energy* **2009**, *83*, 1499-1508, doi:https://doi.org/10.1016/j.solener.2009.04.008.
4. Çörekçi, Ş.; Kizilkaya, K.; Asar, T.; Öztürk, M.; Çakmak, M.; Ozcelik, S. Effects of Thermal Annealing and Film Thickness on the Structural and Morphological Properties of Titanium Dioxide Films. *Acta Physica Polonica A* **2012**, *121*, doi:10.12693/APhysPolA.121.247.
5. Chandra Sekhar, M.; Kondaiah, P.; Jagadeesh Chandra, S.V.; Mohan Rao, G.; Uthanna, S. Substrate temperature influenced physical properties of silicon MOS devices with TiO2 gate dielectric. *Surface and Interface Analysis* **2012**, *44*, 1299-1304, doi:10.1002/sia.5024.
6. Anderson, D.P.; Adnan, R.H.; Alvino, J.F.; Shipper, O.; Donoeva, B.; Ruzicka, J.-Y.; Al Qahtani, H.; Harris, H.H.; Cowie, B.; Aitken, J.B. Gold Clusters Deposited and Activated on Titania. Part II. *Physical chemistry chemical physics* **2013**, *15*, 14806-14813.
7. Anderson, D.P.; Alvino, J.F.; Gentleman, A.; Al Qahtani, H.; Thomsen, L.; Polson, M.I.; Metha, G.F.; Golovko, V.B.; Andersson, G.G. Chemically-Synthesised, Atomically-Precise Gold Clusters Deposited and Activated on Titania. *Physical chemistry chemical physics* **2013**, *15*, 3917-3929.
8. Al Qahtani, H.S.; Metha, G.F.; Walsh, R.B.; Golovko, V.B.; Andersson, G.G.; Nakayama, T. Aggregation Behavior of Ligand-Protected Au9 Clusters on Sputtered Atomic Layer Deposition TiO2. *The Journal of Physical Chemistry C* **2017**, *121*, 10781-10789, doi:10.1021/acs.jpcc.6b11590.
9. Krishnan, G.; Al Qahtani, H.S.; Li, J.; Yin, Y.; Eom, N.; Golovko, V.B.; Metha, G.F.; Andersson, G.G. Investigation of Ligand-Stabilized Gold Clusters on Defect-Rich Titania. *The Journal of Physical Chemistry C* **2017**, *121*, 28007-28016, doi:10.1021/acs.jpcc.7b09514.
10. Wilcoxon, J.P.; Provencio, P. Etching and Aging Effects in Nanosize Au Clusters Investigated Using High-Resolution Size-Exclusion Chromatography. *The Journal of Physical Chemistry B* **2003**, *107*, 12949-12957, doi:10.1021/jp027575y.
11. Moulder, J.F. Handbook of x-ray photoelectron spectroscopy : a reference book of standard data for use in x-ray photoelectron spectroscopy. *Handbook of x-ray photoelectron spectroscopy :* **1992**.
12. Alotabi, A.S.; Gibson, C.T.; Metha, G.F.; Andersson, G.G. Investigation of the Diffusion of Cr2O3 into Different Phases of TiO2 upon Annealing. *ACS Applied Energy Materials* **2021**, *4*, 322-330, doi:10.1021/acsaem.0c02270.
13. Biesinger, M.C.; Brown, C.; Mycroft, J.R.; Davidson, R.D.; McIntyre, N.S. X-ray photoelectron spectroscopy studies of chromium compounds. *Surface and Interface Analysis* **2004**, *36*, 1550-1563, doi:10.1002/sia.1983.
14. Kawawaki, T.; Kataoka, Y.; Hirata, M.; Akinaga, Y.; Takahata, R.; Wakamatsu, K.; Fujiki, Y.; Kataoka, M.; Kikkawa, S.; Alotabi, A.S.; et al. Creation of High-Performance Heterogeneous Photocatalysts by Controlling Ligand Desorption and Particle Size of Gold Nanocluster. *Angewandte Chemie International Edition* **2021**, *60*, 21340-21350, doi:https://doi.org/10.1002/anie.202104911.
